# Phenotypic Profiling of Selected Cellulolytic Strains to Develop a Crop Residue-Decomposing Bacterial Consortium

**DOI:** 10.3390/microorganisms13010193

**Published:** 2025-01-17

**Authors:** Arman Shamshitov, Egidija Satkevičiūtė, Francesca Decorosi, Carlo Viti, Skaidrė Supronienė

**Affiliations:** 1 Microbiology Laboratory, Lithuanian Research Centre for Agriculture and Forestry, Institute of Agriculture, Instituto al. 1, Akademija, LT-58344 Kedainiai, Lithuania; egidija.satkeviciute@lammc.lt; 2 Genexpress Laboratory, Department of Agronomy, Food, Environmental and Forestry (DAGRI), University of Florence, Via della Lastruccia 14, I-50019 Sesto Fiorentino, Italy; francesca.decorosi@unifi.it (F.D.); carlo.viti@unifi.it (C.V.)

**Keywords:** cellulolytic bacteria, straw, decomposition, phenotype microarray, consortium

## Abstract

Slow decomposition rates of cereal crop residues can lead to agronomic challenges, such as nutrient immobilization, delayed soil warming, and increased pest pressures. In this regard, microbial inoculation with efficient strains offers a viable and eco-friendly solution to accelerating the decomposition process of crop residues. However, this solution often focuses mostly on selecting microorganisms based on the appropriate enzymic capabilities and neglects the metabolic versatility required to utilize both structural and non-structural components of residues. Therefore, this study aimed to address these limitations by assessing the metabolic profiles of five previously identified cellulolytic bacterial strains, including *Bacillus pumilus* 1G17, *Micromonospora chalcea* 1G49, *Bacillus mobilis* 5G17, *Streptomyces canus* 1TG5, and *Streptomyces achromogenes* 3TG21 using Biolog Phenotype Microarray analysis. Moreover, this study evaluated the impact of wheat straw inoculation with single strains and a bacterial consortium on soil organic carbon and nitrogen content in a pot experiment. Results revealed that, beyond the core subset of 12 carbon sources, the strains exhibited diverse metabolic capacities in utilizing 106 carbon sources. All strains demonstrated effective straw biomass degradation compared to the negative control, with significant differences detected only in oil seed rape straw biodegradation estimations. Furthermore, wheat straw inoculated with a bacterial consortium showed a significant increase in soil organic carbon content after 180 days in the pot experiment. Overall, these findings underscore the critical role of metabolic profiling in gaining a deeper understanding of microbial capabilities and addressing the complexities of residue composition and environmental variability.

## 1. Introduction

The significant rise in global cereal production, driven by the need to sustain a growing population, has led to a corresponding increase in cereal crop residue generation [1]. In Northern Europe, annual residue production has grown by approximately 17% from 2001 to 2021, while in Lithuania, this increase is even more pronounced, estimated at 75.4% due to expanding cultivation areas and advancements in production technologies [2]. Incorporating crop residues into the soil or leaving them on the soil surface are cost-effective yet essential management practices that promote soil health and the sustainability of agroecosystems [3,4,5,6]. Notably, the return of straw to fields has been shown to significantly increase soil organic carbon levels, as evidenced by a meta-analysis that included 176 experimental studies [7]. Moreover, crop residue decomposition plays a critical role in nutrient cycling, providing essential nutrients to support soil microbial activity and plant growth [8,9].

However, the recalcitrant nature of certain crop residues, particularly those derived from cereal crops, presents challenges for efficient decomposition [2,10]. Cereal crop residues often have a high carbon-to-nitrogen (C/N) ratio compared to residues from other crop types (e.g., *Brassica*), and contain substantial lignocellulose structures, resulting in slower decomposition rates [11]. Under field conditions, slow decomposition can lead to several agronomic issues, such as nutrient immobilization [12,13], increased pest and disease risks [14,15,16], delayed soil warming [17,18], and possible interference with planting, resulting in uneven seedbeds [19].

To address these challenges, microbial inoculation has emerged as a promising strategy for accelerating residue decomposition [20,21,22]. The screening and selection of microorganisms for this purpose typically focus on enzymatic capabilities to degrade lignocellulosic biopolymers, including cellulose, hemicellulose, and lignin [23,24,25,26,27,28,29,30,31]. While these qualitative and quantitative approaches provide critical insights, they sometimes neglect the complex and heterogeneous nature of crop residues. In addition to lignocellulosic structures, crop residues contain a diverse array of organic compounds, including carbohydrates, organic acids, lipids, and secondary metabolites, which also influence decomposition dynamics [32,33,34]. In natural conditions, inoculated microorganisms may utilize various carbon sources for metabolism and exhibit diverse responses to the structural complexity and accessibility of substrates [35]. These factors contribute to the variability in microbial performance, particularly in field settings. Consequently, studies on the limitations and opportunities of soil microbial inoculants emphasize the necessity for more integrated and rational approaches to strain selection, including advanced phenotypic characteristics of microorganisms [36]. Moreover, recent studies also advocate for the use of multi-strain consortia that complement each other’s metabolic capabilities, enabling the utilization of a wider range of substrates and carbon sources, thereby increasing efficacy compared to single-strain inoculants [37,38].

The majority of current studies focus on enzymatic profiling for selecting microbial strains to enhance crop residue decomposition [35,39,40]. While these approaches provide valuable insights into lignocellulose-degrading capabilities, they often overlook the broader functional versatility of microorganisms required to metabolize non-structural components, which is essential for efficient crop residue decomposition. In this context, the Biolog Phenotype Microarray (PM) assay offers a high-throughput platform for profiling the functional diversity of microorganisms by testing their ability to metabolize a wide range of sources and adapt to various conditions [41,42]. This approach complements conventional lignocellulolytic enzyme-centric screening methods by providing a more holistic understanding of microbial functionality. Therefore, this study aimed to assess the metabolic profiles of five bacterial strains selected from 64 cellulolytic strains previously identified and characterized [43]. In addition to evaluating the metabolic versatility of these strains using the Biolog PM assay, this study investigated their ability to degrade straw biomass in a controlled laboratory setup. Furthermore, it examined the impact of wheat straw inoculation, using both single strains and a bacterial consortium, on soil organic carbon and nitrogen content in the pot experiment.

## 2. Materials and Methods

### 2.1. Phenotype Microarray

From sixty-four previously characterized cellulolytic bacterial strains [43], the five most efficient Gram-positive strains were selected for further assessment based on prior analysis. These strains included *Bacillus pumilus* 1G17, *Micromonospora chalcea* 1G49, *Bacillus mobilis* 5G17, *Streptomyces canus* 1TG5, and *Streptomyces achromogenes* 3TG21. To comprehensively assess their metabolic capabilities, the selected strains were analyzed using PM plates (Biolog, Hayward, CA, USA): PM1-2 for carbon source utilization and PM10 for pH tolerance. The full list of compounds assayed in these PMs can be accessed at https://www.biolog.com/wp-content/uploads/2024/06/00A-042-Rev-E-Phenotype-MicroArrays-1-10-Plate-Maps.pdf (accessed on 1 November 2023). Bacterial strains were cultured on carboxymethyl cellulose (CMC) agar medium and incubated at 30 °C for 48 h. Post incubation, colonies were carefully picked using a sterile swab and transferred to a sterile capped tube containing 20 mL of 1×IF-0a (Biolog, Hayward, CA, USA) solution. The cell suspension was stirred with the swab to ensure a uniform distribution of cells. The optical density (OD600) of the suspension was then adjusted to 0.08. Appropriate filter-sterilized 12× PM additive solutions were prepared for PM1 and PM2 (10 mL 240 mM MgCl_2_·6H_2_O and 120 mM CaCl_2_·2H_2_O, 10 mL 3 mM L-arginine, HCl, and 6 mM L-glutamate, Na, 10 mL 0.6% yeast extract, 10 mL 0.6% Tween 80, 30 mL d.d. H_2_O), and for PM10 (10 mL 240 mM MgCl_2_·6H_2_O and 120 mM CaCl_2_·2H_2_O, 10 mL 0.6% yeast extract, 10 mL 0.6% Tween 80, 10mL 300 mM D-glucose, and 600 mM pyruvate Na, 30 mL d.d. H_2_O). The inoculation fluids for PM1 and PM2 plates were prepared by combining 20 mL of IF-0a, 2 mL of dye mix F (Biolog, Hayward, CA, USA), 2 mL of the appropriate 12× PM additive solution, and 1.76 mL of cell suspension. For PM10, 11 mL of IF-10b (Biolog, Hayward, CA, USA), 0.132 mL of dye mix F, 1.1 mL of the appropriate 12× PM additive solution, and 0.968 mL of cell suspension were used. The Phenotype Microarray plates were inoculated with 100 µL of the inoculation fluid per well and incubated in an OmniLog system at 30 °C for 72 h. Readings were recorded every 15 min for over 72 h, and the data were analyzed using OmniLog-PM software version 2.3.01 (Biolog), which generated time-course curves for tetrazolium color formation. Each experiment was conducted in triplicate for PM1–2 and in duplicate for PM10. Data from the OmniLog-PM software version 2.3.01 (release OM_PM_109M) were filtered based on the area of the kinetic curves and transferred to Excel spreadsheets version 2412 (Microsoft Corporation, Albuquerque, NM, USA). For each carbon source, the average area was calculated (*n* = 3). A carbon source was considered utilized by a strain if the average area was at least 50% higher than that of the negative control (A01 well). PM data were plotted using the *ggplot2* and *virdis* packages in “R” version 4.3.2.

### 2.2. Assessment of Temperature Influence on Cellulase Activity

To assess the effect of temperature variations typical of natural conditions on cellulase activity, bacterial strains were incubated on CMC agar for five days at six different temperatures: 5 °C, 10 °C, 15 °C, 20 °C, 25 °C, and 30 °C. Cellulase activity was determined using 1% (*w*/*v*) Congo red as an indicator, following the methodology described in a previous study [43]. The activity was calculated as the ratio of the clear zone diameter to the colony diameter. The presence of a clear zone around bacterial colonies indicated cellulose degradation and served as a proxy for cellulase activity.

### 2.3. Estimation of Crop Residue Biodegradation

A gravimetric method was used to estimate the efficiency of crop residue biodegradation by bacterial strains. For the preparation of bacterial inoculum, the selected bacterial strains were cultured in LB media and incubated in a shaker incubator EXCELLA E24 (Eppendorf, Hamburg, Germany) at 150 rpm and 30 °C for 72 h. Following incubation, the cultures were centrifuged at 3000 rpm for 10 min. The resulting pellet was washed and resuspended in 0.8% (*w*/*v*) NaCl solution. The cell density (OD600) was measured using a biophotometer (Eppendorf, Hamburg, Germany) and adjusted to an OD600 of 0.1 to ensure consistent inoculum concentrations.

The crop residues, including wheat straw (total carbon content of 42.45% d.m.; total nitrogen content of 0.38% d.m) and oilseed rape straw (total carbon content of 44.62% d.m.; total nitrogen content of 0.85% d.m) were ground to a fine powder (0.1 mm) and autoclaved to ensure sterility. Subsequently, 9 mL of autoclaved M9 minimal medium (200 mL/L M9 minimal salts (5× concentration), 2 mL/L 1 M magnesium sulfate, 0.1 mL/L 1 M calcium chloride, and 800 mL/L d.d. H_2_O), supplemented with 100 mg of wheat straw and oilseed rape straw as the carbon source, were inoculated with 1 mL bacterial strain inoculum (*n* = 5). To account for potential non-biological degradation of crop residues, a negative control where 1 mL of d.d. H_2_O was used instead of inoculum was included. This control served as a baseline measurement of abiotic mass loss. The inoculated cultures in tubes were incubated in the shaker incubator at 150 rpm and 30 °C for 7 days (168 h). Following the incubation period, the culture broths were filtered through pre-weighted Whatman No. 1 filter papers to collect the residual biomass. The filter papers containing the residues were dried in a hot air oven at 70 °C for an hour and then reweighed to determine the final mass of the crop residues. The total mass reduction in the crop residues, including wheat straw and oilseed rape straw, was calculated as the difference between the initial mass (*IM*) and the final mass (*FM*) after incubation. The percentage reduction in total mass (*MR*%) was calculated using the following equation:

(1) $$MR\%=\frac{IM-FM}{IM}\times 100$$

The mass loss observed in the negative control was subtracted from the total mass loss observed in the inoculated samples to isolate the actual biodegradation due to bacterial activity.

### 2.4. Pot Experiment

Surface soil samples were collected from the upper 20 cm layer at the experimental site located at the Institute of Agriculture, LAMMC, in the Kedainiai district, Akademija (55°23′50″ N and 23°51′40″ E). According to the World Reference Base for Soil Resources (WRB) classification system, this soil is classified as *Endo-Calcaric–Endohypogleyic Cambisol*. To assess the physicochemical properties of the initial soil, a portion of the collected samples were air-dried and sieved through a 2 mm mesh. Subsequent analyses were conducted using standard analytical techniques, with results detailed in Table 1. Soil pH in KCl (pHKCl) was measured in a 1 N KCl extract using the potentiometric method. The content of plant-available phosphorus and potassium was determined following the Egner–Riehm–Domingo (A-L) method. Organic carbon content was quantified using the dry combustion method. Organic matter was evaluated in accordance with EN 13039:1999 [44], and total nitrogen was measured following EN 13342:2000 [45] using a nitrogen distillation apparatus. Wheat straw was harvested from the same field experiment as the soil samples. The straw was air-dried, chopped into 1–2 cm pieces, and sterilized by autoclaving at 121 °C for 15 min to eliminate potential microbial contaminants. The composition of the wheat straw was determined, with a total carbon content of 42.45% d.m. and a total nitrogen content of 0.38% d.m.

The experiment was conducted in pots (17 cm (diameter) × 13.5 cm (height)) under non-sterile, controlled conditions using a growth room chamber. The growth chambers were maintained with a day/night temperature cycle of 22/16 °C, a light regime of 16 h of daylight followed by 8 h of darkness, a light intensity of 350 μmol m^−2^ s^−1^, and a relative humidity set at 75%. Each pot in the experiment was prepared with a precise mixture consisting of 500 g of sieved, non-sterile soil to evaluate the performance of the bacterial strains in a more ecologically realistic setting, and 10 g of chopped wheat straw sterilized by autoclaving, with prior inoculation with 25 mL of bacterial inoculum. The bacterial inoculum was prepared following the protocol described in Section 2.3. The experimental design included six treatments, each corresponding to a different bacterial strain inoculum: *Bacillus pumilus* 1G17, *Micromonospora chalcea* 1G49, *Bacillus mobilis* 5G17, *Streptomyces canus* 1TG5, *Streptomyces achromogenes* 3TG21, and consortium treatment combining all strains (1G17 + 1G49 + 5G17 + 1TG5 + 3TG21) in an equal ratio. Before preparing the inoculum from the bacterial consortium, strains were tested for compatibility using the co-culturing method on CMC agar plates, which did not reveal any trace of growth inhibition between strains. An inoculum concentration OD600 of 0.1 was used. The effects of these bacterial treatments were compared against two control conditions: soil substrate amended with wheat straw but without bacterial inoculation (C1), and soil substrate without any amendments (C2), with each control receiving 25 milliliters of water. To ensure statistical robustness, each treatment was replicated five times, with the replicates arranged according to a completely randomized design. The experiment was conducted over a period of 180 days, during which pots were watered twice weekly with 30 mL of tap water per watering session, amounting to 60 mL per week. This watering regimen was carefully maintained to ensure optimal moisture conditions. Organic carbon and total nitrogen content in the soil substrate from the pots were monitored after 90 and 180 days according to EN 13039:1999 and EN 13342:2000, respectively.

### 2.5. Statistical Analysis

The data from the estimation of crop residue biodegradation and the pot experiment were tested for normality and homogeneity using the Shapiro–Wilk test and Levene’s test, respectively. For datasets meeting both assumptions, analysis of variance (ANOVA) was performed, followed by Tukey’s HSD test for post hoc comparisons. For datasets that did not meet the assumptions of normality or homogeneity, the Kruskal–Wallis test was applied, with Dunn’s test used for post hoc analysis. All statistical tests were performed using “R” version 4.3.2.

## 3. Results

### 3.1. Phenotype Microarray Analysis of Carbon Sources Utilization and pH Sensitivity

**Figure 1:**
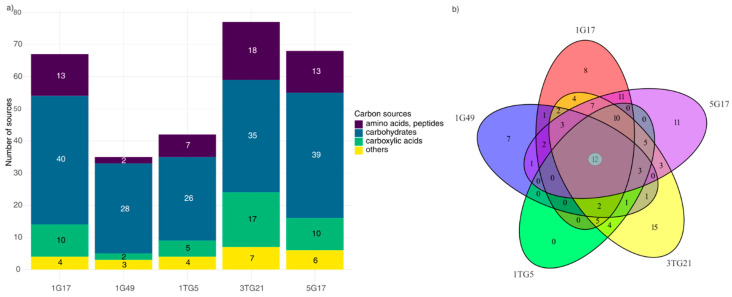
(**a**) Number of carbon sources used by bacterial strains. (**b**) Venn diagram showing the unique or similar capability of carbon source utilization. Colors represent strains: red (1G17), blue (1G49), green (1TG5), yellow (3TG21), and purple (5G17). Numbers indicate unique or shared features, with “12” at the center representing carbon sources utilized by all strains.

PMs were used to systematically assess and screen the metabolic versatility and pH tolerance of the selected bacterial strains. The metabolic ability of these strains to utilize 190 distinct carbon sources, including carbohydrates, carboxylic acids, alcohols, amides, amines, esters, fatty acids, and polymers, was analyzed using PM1–2 plates (Biolog). A core subset of 12 carbon sources was commonly metabolized by all strains, including one amino acid (L-glutamic acid) out of 27 tested, 10 carbohydrates out of 93, and 1 carbon source (gelatin) out of 19 others (Figure 1a,b). Beyond this core subset, the strains displayed varied metabolic capacities of utilizing 106 carbon sources, while 72 remained unutilized by any strain. Strain 1G49 demonstrated the lowest metabolic capacity, using only 35 substrates (18.4%), while strain 3TG21 showed greater ability, metabolizing 77 (40.5%) of the tested carbon sources. Hence, the utilization of compound classes also differed, with strains metabolizing between 6.5% and 41.9% of amino acids and peptides, 27.7% to 42.6% of carbohydrates, and 4.3% to 37% of carboxylic acids.

**Figure 2:**
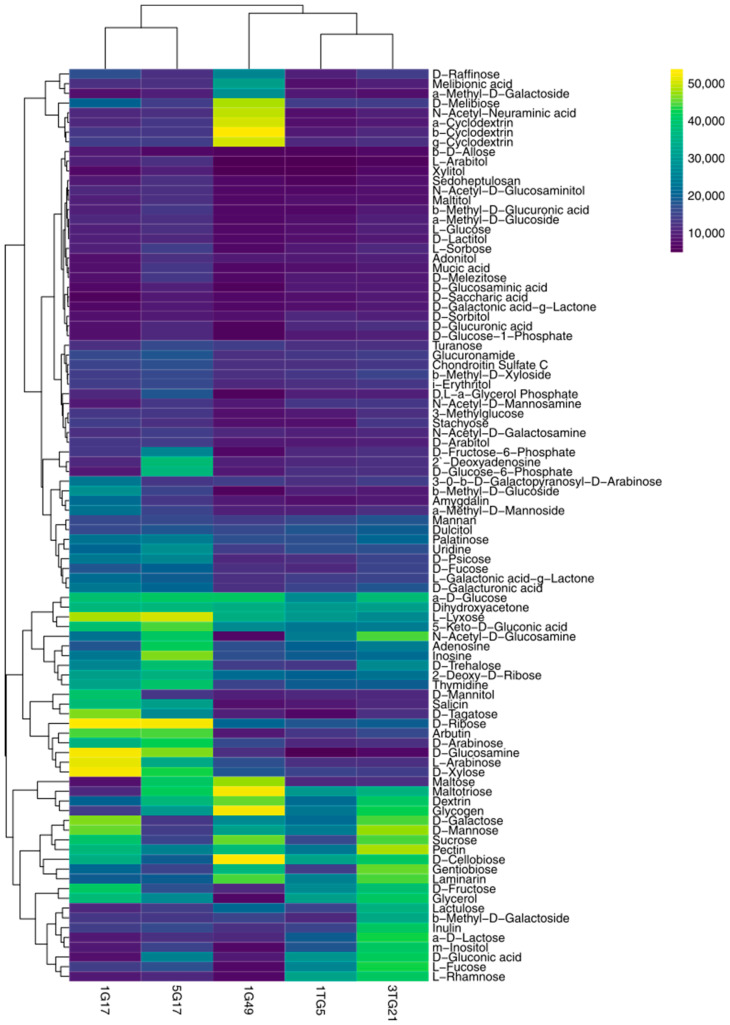
Heat map displaying the phenotypic utilization profiles of bacterial strains across various carbohydrate sources, as assessed using PM 1–2 carbon sources. Data are expressed in arbitrary OmniLog units (AOUs) and represent the area under the kinetic curves after 60 h of incubation in the OmniLog instrument at 30 °C. The color gradient ranges from purple to yellow, with purple indicating a low metabolic response or source utilization, and yellow indicating a high metabolic response or source utilization.

Distinct carbohydrate utilization patterns provided valuable insights into the metabolic potential of these strains for lignocellulose degradation (Figure 2). Clustering analysis using Euclidean distance revealed correlations between metabolic profiles and taxonomic attributes, notably grouping related strains, such as *Bacillus pumilus* 1G17 with *Bacillus mobilis* 5G17 and *Streptomyces canus* 1TG5 with *Streptomyces achromogenes* 3TG21 (Figure 2). The *Bacillus* strains, 1G17 and 5G17, demonstrated broader carbohydrate utilization, metabolizing 42.6% and 41.5% of the tested carbohydrates, respectively, compared to other strains. All strains universally metabolized monosaccharides like D-ribose, α-D-glucose, and dihydroxyacetone, while D-xylose was utilized only by 1G17, 1G49, and 5G17. Notably, 1G49 was the sole strain capable of metabolizing the amino sugar N-acetyl-neuraminic acid. On the other hand, D-cellobiose was consistently used by all bacterial strains. Several glycoside carbohydrates, including 3-0-β-D-galactopyranosyl-D-arabinose, melibionic acid, α-methyl-D-galactoside, β-methyl-D-glucoside, 2-deoxyadenosine, amygdalin, β-methyl-D-galactoside, and α-methyl-D-mannoside, were exclusively utilized by only one of the bacterial strains. Moreover, among the ten polysaccharides tested, all bacterial strains exhibited metabolic activity on dextrin, laminarin, and pectin utilization. However, α-cyclodextrin, β-cyclodextrin, and γ-cyclodextrin were uniquely metabolized by 1G49, while inulin and mannan were exclusively metabolized by 3TG21.

The PM analysis showed that L-glutamic acid was the only amino acid utilized by all bacterial strains (Figure S1). In contrast, seven other amino acids were exclusively metabolized by specific strains, while eight amino acids were not utilized by any strain. Among the amines and amides, only putrescine was metabolized, uniquely by strain 3TG21, with the remaining six compounds in this category unused by any strain (Figure S2). In terms of carboxylic acids, the strains displayed variable metabolic capabilities, utilizing 21 of the tested compounds (Figure S3). Notably, 11 of these acids were exclusively metabolized by specific strains, while 25 were not utilized by any strain. Strains 1G17, 1TG5, and 3TG21 shared the ability to use both succinic acid and citric acid. It was noted that 1G49 exhibited a narrower metabolic profile, being able to utilize only methylpyruvate and pyruvic acid in this category of carbohydrates.

**Figure 3:**
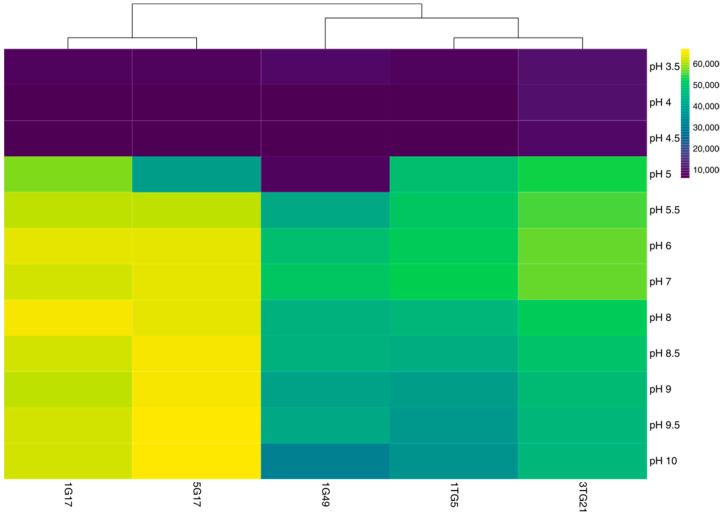
Heat map illustrating the pH sensitivity profiles of bacterial strains, analyzed using PM 10 (wells A1–A12). Data are quantified in arbitrary OmniLog units (AOUs), representing the area under the kinetic growth curves following 60 h of incubation at 30 °C in the OmniLog instrument. The color gradient ranges from purple to yellow, with purple indicating a low metabolic response, and yellow indicating a high metabolic response.

The PM analysis of pH sensitivity across five bacterial strains revealed similarities and differences in their metabolic activity responses to varying pH levels (3.5–10), as illustrated in the heatmap (Figure 3 and Figure S4). Under highly acidic conditions (pH 3.5 to pH 4.5), metabolic activity was uniformly low across all strains but began to increase at pH 5.0–5.5. Notably, strain 1G49 exhibited minimal tolerance at pH 5, exhibiting low metabolic activity at this level. All strains demonstrated peak metabolic activity within the neutral pH range (pH 6–7). However, as the pH shifted into the alkaline range (pH 8.5–10), a moderate decline in metabolic activity was observed in strains 1G49, 1TG5, and 3TG21. In contrast, strains 1G17 and 5G17 maintained relatively high metabolic activity, even at pH levels as high as 10.

**Figure 4:**
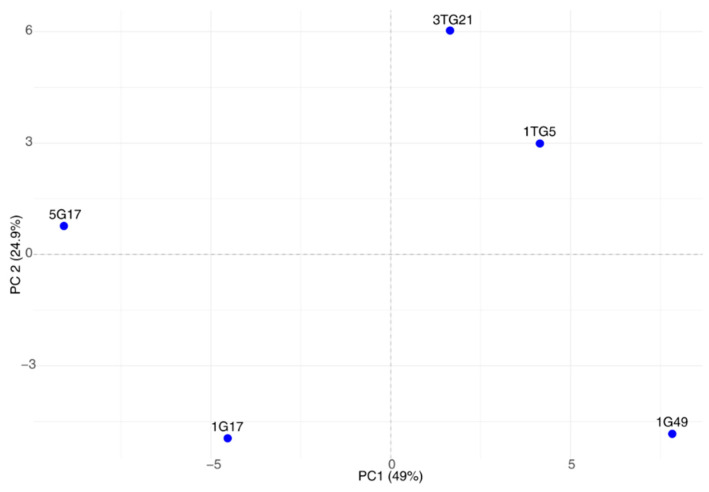
Principal component analysis of PM 1–2 and PM 10 data.

Principal component analysis (PCA) of the Phenotype Microarray data revealed distinct clustering patterns among bacterial strains based on their metabolic profiles (Figure 4). The first principal component (PC1) accounted for 49.02% of the variance, while the second principal component (PC2) explained an additional 24.93%. Strains 1G17 and 1G49 displayed opposite loadings along PC1, emphasizing their distinct metabolic preferences. Strain 5G17 clustered closer to 1G17 along PC1 but exhibited opposite loadings along PC2, indicating both an overlap and a divergence in metabolic behavior. Meanwhile, strains 1TG5 and 3TG21 were positioned relatively close to each other yet were distinctly separated from the other strains in the two-dimensional space, highlighting their unique metabolic profiles.

### 3.2. Effect of Temperature on Cellulolytic Activity

**Figure 5:**
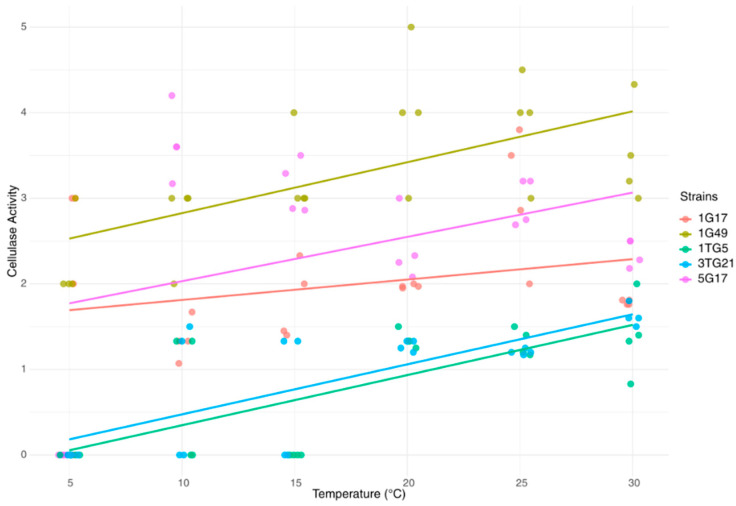
The relationship between temperature (°C) and cellulase activity of bacterial strains. Data points are color-coded by strain, and linear regression trends highlight the activity patterns as temperature changes.

Figure 5 illustrates the relationship between temperature and cellulase activity in bacterial strains. The trends observed in the results reveal a generally positive correlation between temperature and cellulase activity for all strains, albeit with varying rates of activity increase. At 5 °C, cellulase activity was detected only in strains 1G17 and 1G49. Across all strains, cellulase activity increased with rising temperatures, suggesting that enzymatic efficiency is temperature-dependent within the experimental range (5 °C to 30 °C). Among the strains, 1G49 demonstrated comparatively higher efficiency overall, while 3TG21 and 1TG5 exhibited the lowest activity levels.

### 3.3. Bacterial Biodegradation Efficiency of Crop Residues

**Figure 6:**
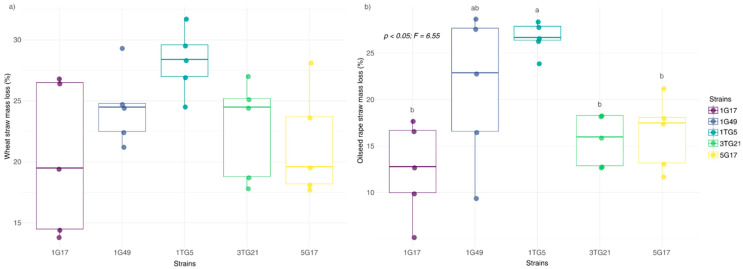
Mass loss (%) of wheat straw (**a**) and oilseed rape straw (**b**) after biodegradation by bacterial strain treatment. Each box plot represents the distribution of five replicate measurements, showing the median, interquartile range, and data range. Compact letter displays above each box plot indicate significant differences between strains (*p* < 0.05) based on Tukey’s HSD test for pairwise comparisons.

A crop straw biodegradation assay was performed to estimate the ability of selected strains to utilize straw as composite biomass, containing complex compounds including lignocellulose matrix. The results demonstrated that all bacterial strains showed significantly higher biodegradation ability of substrates compared to the negative control (wheat straw: 4.20% ± 0.95% mass loss; oilseed rape straw: 4.92% ± 0.89% mass loss), with these baseline values subtracted from the mass loss of inoculated samples for a more accurate assessment. After a seven-day incubation period, a relatively high average biomass loss was recorded for wheat straw at 23.45% ± 4.85%, followed by oilseed rape straw at 18.46% ± 6.70% (Figure 6). No significant differences in wheat straw degradation efficiency were observed among the tested bacterial strains (*p* = 0.065), with mass loss ranging from 20.26% ± 6.7% for strain 1G17 to 28.28% ± 2.71% for strain 1TG5 (Figure 6a). In contrast, oilseed rape straw was degraded less efficiently than wheat straw, with significant differences in mass loss observed among the strains (*p* = 0.002) (Figure 6b). Pairwise comparisons revealed that strain 1TG5 significantly enhanced the degradation rate compared to other strains, except for the strain 1G49.

### 3.4. Effect of Wheat Straw Bacterial Inoculation on Organic Carbon and Total Nitrogen in Soil Substrate

**Figure 7:**
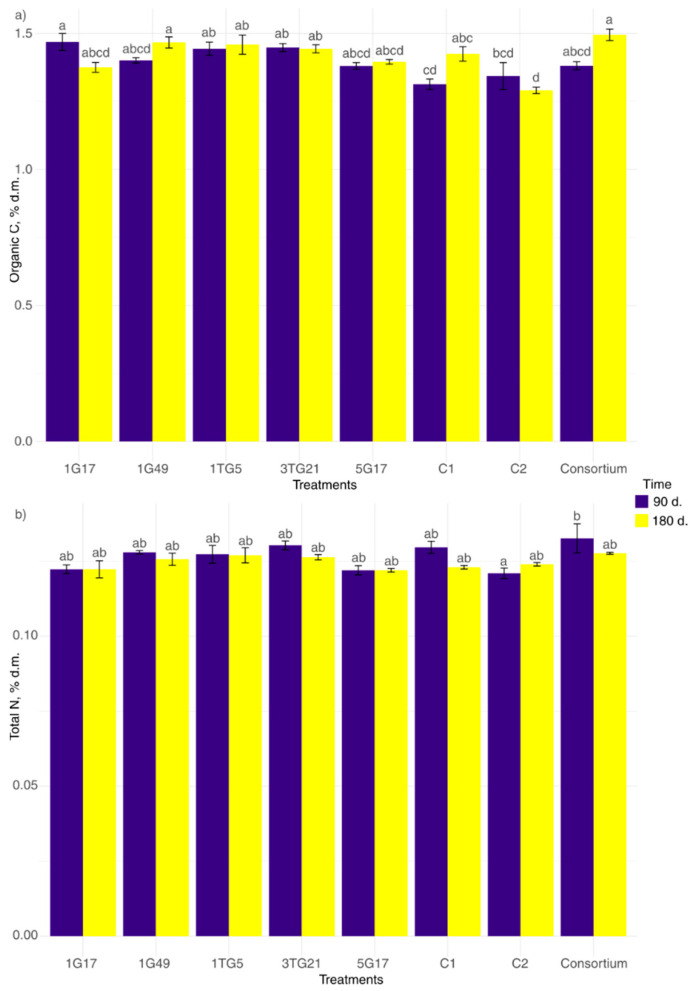
Changes in organic carbon (**a**) and total nitrogen (**b**) contents in soil substrate as a result of wheat straw bacterial inoculation treatments over two time points: 90 days (purple) and 180 days (yellow). C1 and C2 refer to soil substrate + wheat straw without inoculation and only soil substrate, respectively. Data points are means of three replicates, with the standard error shown by error bars. Compact letter displays above bars indicate significant differences between treatments at *p* < 0.05, as determined by Tukey’s HSD test.

The pot experiment results indicate that the application of different bacterial inoculum treatments to wheat straw significantly influenced the organic carbon content in soil substrate (*p* < 0.001) (Figure 7a). Moreover, there was a significant interaction between treatment and time (*p* < 0.001), while the effect of time alone was not statistically significant (*p* = 0.0759). After 90 days, the lowest organic carbon content was observed in the C1 (soil substrate + wheat straw without inoculation) treatment (1.34% ± 0.08%), whereas the 1G17 treatment (1.47% ± 0.05%) displayed the highest organic carbon content in the soil substrate. After 180 days, changes in organic carbon content were observed in response to several treatments. The highest organic carbon content was observed in the soil substrate where straw was inoculated with bacterial consortium (1.50% ± 0.04%), while the lowest value was noted in the C2 (soil substrate-only) treatment (1.38% ± 0.03%). Certain bacterial inoculum treatments, including 1TG5, 3TG21, and 5G17, showed no changes, maintaining a stable organic carbon level in soil substrate throughout the experimental period.

Figure 7b illustrates the changes in soil substrate total nitrogen content in response to different bacterial inoculum treatments of wheat straw across two time periods: 90 days and 180 days. The two-way ANOVA results reveal a significant main effect of treatment on total nitrogen content in soil substrate (*p* = 0.001), indicating that different treatments had a distinct impact on total nitrogen levels. However, the effect of time alone (*p* = 0.069) and the interaction between treatment and time (*p* = 0.326) were not statistically significant. Pairwise comparison showed differences only after 90 days, particularly between the consortium and C2 treatments. A general trend of a decrease in total nitrogen content in soil substrate from 90 days to 180 days, regardless of the bacterial inoculum treatments applied, was observed.

## 4. Discussion

Plant residues comprise a diverse array of simple and complex carbon sources, contributing to both structural and non-structural components that influence their decomposition dynamics [46]. Hence, understanding the metabolic capabilities of microorganisms involved in this process, particularly their ability to utilize a broad spectrum of carbon sources, is therefore critical for identifying and optimizing microbial candidates to enhance decomposition and support sustainable agricultural residue management. To gain insight into the capabilities of bacterial strains, PMs were used to comprehensively evaluate the metabolic capacity and pH tolerance of selected bacterial strains.

The presence of certain carbon sources such as D-cellobiose, dextrin, pectin, and some of the monosaccharides within the core subset highlights that all studied bacterial strains possess the essential metabolic capabilities required for plant residue decomposition. For instance, D-cellobiose, a disaccharide derived from cellulose hydrolysis [47,48], reflects the strains’ ability to target cellulose, a major structural component of plant cell walls [26,49]. The utilization of D-cellobiose indicates the presence of beta-glucosidase activity [50,51,52], which enables the breakdown of cellulose into simpler sugars for further metabolism. Similarly, the simultaneous utilization of the complex polysaccharide pectin, a key component of plant cell walls and the middle lamella, underscores their potential ability to secrete pectinase enzymes, which catalyze the hydrolysis of pectin, facilitating its degradation [53,54].

It is important to note that ferulic acid, although less studied, can hinder microbial lignocellulose degradation by linking lignin and other plant cell polymers via ester and ether bonds, contributing to the structural integrity of the plant cell wall [55]. Wheat, like other cereal crops classified as commelinoid monocotyledons, possesses a type II cell wall characterized by the abundance of glucuronoarabinoxylan, a polysaccharide consisting of a xylan backbone with branches of arabinose and glucuronic acid. Arabinose residues can esterify with feruloyl and p-coumaroyl groups, facilitating oxidative cross-linking of glucuronoarabinoxylan. Consequently, wheat straw, like other commelinoids, contains significant amounts of ferulic acid-branched glucuronoarabinoxylan [56,57,58]. In this study, the absence of ferulic acid in PM 1–2 does not clarify whether the strains can directly utilize these compounds. However, related L-arabinose and D-arabinose were exclusively utilized by 1G17 and 5G17 (both *Bacillus* sp.), while 3-0-b-D-galactopyranosyl-D-arabinose was utilized only by 1G17. Additionally, previous studies indicate that feruloyl esterase enzymes, which cleave ester or ether bonds between ferulic acid and polysaccharides in plant cell walls, exhibit synergistic activity with other lignocellulolytic enzymes [59,60]. This enzyme production was detected in several *Bacillus* spp. including *B. pumilus* SK52.001 [60], *B. pumilus* W3 [61], *B. amyloliquefaciens* H47, and *B. subtilis* WB600 [62], as well as in *Streptomyces* spp. [63] such as *S. cinnamoneus* NBRC 1285 [64] and *S. ambofaciens* [65].

Furthermore, the strains 1G17, 1G49, and 5G17 were able to use the monosaccharide D-xylose, a hemicellulosic sugar that is relatively abundant in agricultural residues [66]. It has been reported that the *xyl* operon, which encodes essential genes, including *xylAB* (xylose metabolism), *xylFGH* (xylose transporter), and *xylR* (xylose regulator), enables the assimilation of D-xylose and its conversion into intermediates for central metabolic pathways [67,68,69]. The recent study on the genome and proteome of *Bacillus coagulans* (B-768) discovered that alongside *xylFGH*, B-768 contains xylose H+-symporter (XYLT, encoded by *xylT*), which is located over 1 Mbp away from *xylA*, *xylB*, and *xylR*, suggesting potentially diverse genetic and regulatory frameworks that bacterial strains employ to metabolize D-xylose [70]. Interestingly, strain 3TG21 exclusively utilized putrescine, a biogenic amine. Putrescine is a precursor in the polyamine biosynthesis pathway and plays a role in the lignification process, and its conjugation with hydroxycinnamic acids forms hydroxycinnamic acid amides, which are incorporated into the lignin matrix to strengthen plant cell walls [71,72,73].

Crop residues contain a wide range of organic compounds, including organic acids that can occur naturally or are predominantly produced through microbial activity during decomposition [74,75]. In the context of microbial consortia associated with crop residue decomposition, the capability of certain species to utilize carboxylic acids produced by others may foster synergistic relationships, thereby enhancing overall decomposition rates. In this study, strain 3TG21 exhibited the highest rate of carboxylic acid utilization (37%), significantly outperforming other strains, which ranged from 4.3% (1G49) to 21.7% (1G17 and 5G17). Moreover, certain carboxylic acids can be key intermediates in central metabolic pathways such as the tricarboxylic acid cycle (TCA), which is essential for energy production and biosynthesis [76,77]. Interestingly, succinic and fumaric acids, integral intermediates of the TCA cycle [78,79,80], were utilized similarly by 1G17, 1TG5, and 3TG21. This shared metabolic trait suggests that these strains may exhibit comparable adaptations for energy generation and biosynthetic processes during decomposition.

The biodegradation assay, using a gravimetric analysis of residual biomass, assessed the ability of previously characterized bacterial strains to degrade crop residues, specifically wheat straw and oilseed rape straw, as primary carbon sources in M9 minimal medium. The results of this assessment demonstrated distinct biodegradation capabilities among the strains for these substrate types.

Typically, the crop residue C/N ratio is a critical determinant of residue quality, influencing microbial degradation rates [33,81]. Higher C/N ratios, as seen in wheat straw with a ratio of 110 in our case, generally impede the degradation process due to limited nitrogen, which is essential for microbial growth and metabolism. In contrast, oilseed rape straw, with a comparatively lower C/N ratio of 52, was expected to support faster degradation. Interestingly, the results showed that wheat straw was more frequently utilized by the bacterial strains than oilseed rape straw (Figure 1). This finding aligns with the study evaluating biomass degradation by *Streptomyces thermocarboxydus*, which demonstrated a higher degradation ratio for cereal straw (barley) compared to canola straw [82]. Additionally, the slower degradation of oilseed rape straw might be attributed to its higher lignin content compared to wheat straw [83] and the presence of secondary metabolites, such as glucosinolates [84], which are known to inhibit microbial activity.

*Bacillus* spp. are extensively studied for their lignocellulolytic enzyme production capabilities, positioning them as leading candidates for biomass conversion [40,85,86,87,88,89]. In this study, both *Bacillus pumilus* 1G17 and *Bacillus mobilis* 5G17 exhibited comparable biodegradation ability among the tested strains; however, neither strain demonstrated the highest efficiency in straw biodegradation. The utilization of *Streptomyces* spp. for lignocellulose biomass conversion has also been well documented [90,91,92,93]. Gong et al. [94] reported that *Streptomyces* spp., after process optimization, achieved a 60.55% weight loss in corn straw after 7 days of incubation. Similarly, another study showed that the SD-1 strain of *Streptomyces griseorubens* significantly enhanced rice straw degradation, resulting in about a 76% weight loss in 30 days, with notable acceleration during the initial stages compared to the control [95]. In our case, *Streptomyces canus* 1TG5 exhibited a slight but non-significant advantage in wheat straw degradation, while the advantage in oilseed rape straw degradation was significant compared to other strains, including *Streptomyces achromogenes* 3TG21, except for *Micromonospora chalcea* 1G49. Among the studied bacteria, *Micromonospora* spp. is less studied for lignocellulose biomass conversion, despite their demonstrated potential for lignocellulolytic enzyme production [96,97,98]. Notably, genome analysis of *Micromonospora* spp. has revealed that the CP22 strain possesses 20 genes encoding cellulases and 40 genes encoding hemicellulases, which positions it as a promising candidate for lignocellulose conversion processes [99].

In our experiment, the inoculation of wheat straw with a bacterial consortium led to an increase in organic carbon levels only after 180 days compared to C2 (soil substrate-only). Similarly, the incorporation of sugarcane straw inoculated with a microbial consortium, consisting of *Chaetomium* sp., *Scytalidium* sp., *Corynascus* sp., *Streptomyces* sp., and *Bacillus* sp., significantly increased soil organic carbon compared to the incorporation of straw without inoculation and control [30]. Although the consortium in this study showed a trend of higher organic carbon with respect to C1 (soil substrate + wheat straw without inoculation), the difference was not statistically significant. Thus, these findings suggest that bacterial activity in the consortium may enhance soil organic carbon levels by supporting native microbial communities, potentially through microbial biomass synthesis or the accumulation of decomposition intermediates. In contrast, a study on bacterial consortium inoculation during rice straw composting reported a significant decrease in organic matter content compared to the control, with effects evident after just 15 days of composting [100]. Zhou et al. [101] reported that co-composting of cow manure (10% and 20% addition) with corn straw inoculated with *Streptomyces*-*Bacillus* significantly increased total nitrogen content during the initial composting stage compared to inoculant-free controls, which can be attributed to microbial accumulation facilitated by the added cow manure. However, during the decomposition of carbon-rich, nitrogen-poor crop residues, as in this study, microorganisms typically require additional nitrogen to build biomass and maintain metabolic activity, which usually results in a reduction in nitrogen in the soil [102,103,104]. Similarly, the study on wheat straw decomposition under different tillage practices demonstrated that increased microbial activity, particularly during the spring and summer seasons, led to nitrogen immobilization from the soil [105].

Even though strains for this study were proven to have cellulolytic and lignocellulolytic activities, it is crucial to acknowledge the observed partial contradiction between the crop residue decomposition results and the findings from the PM analysis. The PM data, which demonstrated the utilization of a broader spectrum of carbon sources by some strains (e.g., 3TG21), should refer to the more efficient biodegradation capacity of crop residues. One of the possible reasons can be explained by the limitations of the gravimetric method, which cannot accurately depict bacterial biodegradation of crop residues due to taking only direct measurements of residue mass loss. However, this method cannot account for the breakdown of structural and non-structural components, which may not result in substantial weight loss but still are critical indicators of biodegradation efficiency. On the other hand, the PM primarily tests individual carbon substrates and cannot fully facilitate the complex structure of crop residues. To bridge these gaps, future studies should investigate microbial inoculation under in situ conditions to better capture “real-world” dynamics. It is also essential to explore how bacterial inoculation influences native soil microbial communities.

## 5. Conclusions

The Biolog PM analysis provided valuable insights into the metabolic profiles of the selected cellulolytic bacterial strains, emphasizing their potential to enhance crop residue decomposition. This study revealed that these strains possess essential metabolic traits for degrading structural components of crop residues, while varying in their ability to utilize non-structural carbon sources commonly present in plant tissue and soil. The microbial consortium constructed from these strains enhanced soil organic carbon levels in the pot experiment, highlighting the advantage of integrating metabolically diverse strains over single-strain inoculations. These findings highlight the importance of metabolic profiling as a tool for understanding microbial capabilities and addressing the complexities of residue composition and environmental variability.

## Figures and Tables

**Table 1:** Physicochemical properties of the initial experimental soil.

Characteristic	Value
Texture	Loam
pHKCl	6.4
P_2_O_5_, mg/kg d.m.	262.1 ± 12.8
K_2_O, mg/kg d.m.	314 ± 17.3
Organic C, % d.m.	1.387 ± 0.086
Total N, % d.m.	0.116 ± 0.002
Organic matter, % d.m.	4.87 ± 0.37

## Data Availability

Data are contained within the article and supplementary materials.

## Supplementary Materials

The following supporting information can be downloaded at: https://www.mdpi.com/article/10.3390/microorganisms13010193/s1, Figure S1: Heat map displaying the phenotypic utilization profiles of amino acid sources, as assessed using PM 1–2 carbon sources. Figure S2: Heat map displaying the phenotypic utilization profiles of other sources, as assessed using PM 1–2 carbon sources. Figure S3: Heat map displaying the phenotypic utilization profiles of five bacterial strains across carboxylic acids, as assessed using Biolog Phenotype Microarray (PM) 1–2 carbon sources. Figure S4: Heat map illustrating the pH sensitivity profiles of bacterial strains, analyzed using PM 10.

## References

[1] Harindintwali J.D., Zhou J., Yu X. (2020). Lignocellulosic Crop Residue Composting by Cellulolytic Nitrogen-Fixing Bacteria: A Novel Tool for Environmental Sustainability. Sci. Total Environ..

[2] Shamshitov A., Kadžienė G., Supronienė S. (2024). The Role of Soil Microbial Consortia in Sustainable Cereal Crop Residue Management. Plants.

[3] Sarkar S., Skalicky M., Hossain A., Brestic M., Saha S., Garai S., Ray K., Brahmachari K. (2020). Management of Crop Residues for Improving Input Use Efficiency and Agricultural Sustainability. Sustainability.

[4] Singh Y., Singh B., Timsina J. (2005). Crop Residue Management for Nutrient Cycling and Improving Soil Productivity in Rice-Based Cropping Systems in the Tropics. Adv. Agron..

[5] Fu B., Chen L., Huang H., Qu P., Wei Z. (2021). Impacts of Crop Residues on Soil Health: A Review. Environ. Pollut. Bioavailab..

[6] Turmel M.S., Speratti A., Baudron F., Verhulst N., Govaerts B. (2015). Crop Residue Management and Soil Health: A Systems Analysis. Agric. Syst..

[7] Liu C., Lu M., Cui J., Li B., Fang C. (2014). Effects of Straw Carbon Input on Carbon Dynamics in Agricultural Soils: A Meta-Analysis. Glob. Change Biol..

[8] Torma S., Vilček J., Lošák T., Kužel S., Martensson A. (2018). Residual Plant Nutrients in Crop Residues–an Important Resource. Acta Agric. Scand. B Soil Plant Sci..

[9] Cavalli E., Lange A., Cavalli C., Behling M. (2018). Decomposition and Release of Nutrients from Crop Residues on Soybean-Maize Cropping Systems. Rev. Bras. Cienc. Agrar..

[10] Ntonta S., Mathew I., Zengeni R., Muchaonyerwa P., Chaplot V. (2022). Crop Residues Differ in Their Decomposition Dynamics: Review of Available Data from World Literature. Geoderma.

[11] Habets S., de Wild P.J., Huijgen W.J.J., van Eck E.R.H. (2013). The Influence of Thermochemical Treatments on the Lignocellulosic Structure of Wheat Straw as Studied by Natural Abundance 13C NMR. Bioresour. Technol..

[12] de Bortolli M.A., Assmann T.S., de Bortolli B.B., Maccari M., Bernardon A., Jamhour J., Franzluebbers A.J., Soares A.B., Severo I.K. (2024). Nutrient Dynamics in Integrated Crop–Livestock Systems: Effects of Stocking Rates and Nitrogen System Fertilization on Litter Decomposition and Release. Agronomy.

[13] Sharma S., Singh P., Choudhary O.P., Neemisha (2021). Nitrogen and Rice Straw Incorporation Impact Nitrogen Use Efficiency, Soil Nitrogen Pools and Enzyme Activity in Rice-Wheat System in North-Western India. Field Crops Res..

[14] Pereyra S., Lori G.A. (2013). Crop Residues and Their Management in the Epidemiology of Fusarium Head Blight. Fusarium Head Blight in Latin America.

[15] Colbach N., Meynard J.M. (1995). Soil Tillage and Eyespot: Influence of Crop Residue Distribution on Disease Development and Infection Cycles. Eur. J. Plant Pathol..

[16] Porcella F., Buhler D.D., McGiffen M.E. (2018). Pest Management and Crop Residues. Crops Residue Management.

[17] Moroizumi T., Horino H. (2002). The Effects of Tillage on Soil Temperature and Soil Water. Soil Sci..

[18] Shen Y., McLaughlin N., Zhang X., Xu M., Liang A. (2018). Effect of Tillage and Crop Residue on Soil Temperature Following Planting for a Black Soil in Northeast China. Sci. Rep..

[19] Wuest S.B., Albrecht S.L., Skirvin K.W. (2000). Crop Residue Position and Interference with Wheat Seedling Development. Soil Tillage Res..

[20] Che S., Xu Y., Qin X., Tian S., Wang J., Zhou X., Cao Z., Wang D., Wu M., Wu Z. (2024). Building Microbial Consortia to Enhance Straw Degradation, Phosphorus Solubilization, and Soil Fertility for Rice Growth. Microb. Cell Fact..

[21] Singh A., Tv A., Singh S., Saxena A.K., Nain L. (2024). Application of Fungal Inoculants Enhances Colonization of Secondary Bacterial Degraders during in Situ Paddy Straw Degradation: A Genomic Insights into Cross-Domain Synergism. Int. Microbiol..

[22] Bhattacharjya S., Sahu A., Phalke D.H., Manna M.C., Thakur J.K., Mandal A., Tripathi A.K., Sheoran P., Choudhary M., Bhowmick A. (2021). In Situ Decomposition of Crop Residues Using Lignocellulolytic Microbial Consortia: A Viable Alternative to Residue Burning. Environ. Sci. Pollut. Res..

[23] Lu W.J., Wang H.T., Yang S.J., Wang Z.C., Nie Y.F. (2006). Isolation and Characterization of Mesophilic Cellulose-Degrading Bacteria from Flower Stalks-Vegetable Waste Co-Composting System. J. Gen. Appl. Microbiol..

[24] Rawway M., Ali S.G., Badawy A.S. (2018). Isolation and Identification of Cellulose Degrading Bacteria from Different Sources at Assiut Governorate (Upper Egypt). J. Ecol. Health Environ..

[25] Kakkar N., Gupta S.K., Singh Saharan B. (2016). Comparison of Different Stains in Determination of Extracellular Cellulose Activity of Odontotermes Obesus Gut Bacteria. Int. J. Entomol. Res..

[26] Pérez J., Muñoz-Dorado J., De La Rubia T., Martínez J. (2002). Biodegradation and Biological Treatments of Cellulose, Hemicellulose and Lignin: An Overview. Int. Microbiol..

[27] Puițel A.C., Suditu G.D., Danu M., Ailiesei G.L., Nechita M.T. (2022). An Experimental Study on the Hot Alkali Extraction of Xylan-Based Hemicelluloses from Wheat Straw and Corn Stalks and Optimization Methods. Polymers.

[28] Huang Z., He C., Wang Z., Luo X., Sun X., Zhao J., Gao X., Xiang W., Song J., Wang X. (2022). *Nocardia rosealba* sp. Nov., a Novel Ligninase-Producing Actinobacterium Isolated from Soil. Int. J. Syst. Evol. Microbiol..

[29] Vasina D.V., Moiseenko K.V., Fedorova T.V., Tyazhelova T.V. (2017). Lignin-Degrading Peroxidases in White-Rot Fungus Trametes Hirsuta 072. Absolute Expression Quantification of Full Multigene Family. PLoS ONE.

[30] Phukongchai W., Kaewpradit W., Rasche F. (2022). Inoculation of Cellulolytic and Ligninolytic Microorganisms Accelerates Decomposition of High C/N and Cellulose Rich Sugarcane Straw in Tropical Sandy Soils. Appl. Soil Ecol..

[31] Sasikumar V., Priya V., Shiv Shankar C., Sathish Sekar D. (2014). Isolation and Preliminary Screening of Lignin Degrading Microbes. J. Acad. Ind. Res. (JAIR).

[32] Horwath W., Paul E.A. (2007). Carbon Cycling and Formation of Soil Organic Matter. Soil Microbiology, Ecology and Biochemistry.

[33] Grzyb A., Wolna-Maruwka A., Niewiadomska A. (2020). Environmental Factors Affecting the Mineralization of Crop Residues. Agronomy.

[34] Kögel-Knabner I. (2002). The Macromolecular Organic Composition of Plant and Microbial Residues as Inputs to Soil Organic Matter. Soil Biol. Biochem..

[35] Wei Y., Wu D., Wei D., Zhao Y., Wu J., Xie X., Zhang R., Wei Z. (2019). Improved Lignocellulose-Degrading Performance during Straw Composting from Diverse Sources with Actinomycetes Inoculation by Regulating the Key Enzyme Activities. Bioresour. Technol..

[36] O’Callaghan M., Ballard R.A., Wright D. (2022). Soil Microbial Inoculants for Sustainable Agriculture: Limitations and Opportunities. Soil Use Manag..

[37] Liu X., Mei S., Salles J.F. (2023). Inoculated Microbial Consortia Perform Better than Single Strains in Living Soil: A Meta-Analysis. Appl. Soil Ecol..

[38] Santos M.S., Nogueira M.A., Hungria M. (2019). Microbial Inoculants: Reviewing the Past, Discussing the Present and Previewing an Outstanding Future for the Use of Beneficial Bacteria in Agriculture. AMB Express.

[39] Sharma R.K., Arora D.S. (2015). Fungal Degradation of Lignocellulosic Residues: An Aspect of Improved Nutritive Quality. Crit. Rev. Microbiol..

[40] Mei J., Shen X., Gang L., Xu H., Wu F., Sheng L. (2020). A Novel Lignin Degradation Bacteria-Bacillus Amyloliquefaciens SL-7 Used to Degrade Straw Lignin Efficiently. Bioresour. Technol..

[41] Shea A., Wolcott M., Daefler S., Rozak D.A. (2012). Biolog Phenotype Microarrays. Methods Mol. Biol..

[42] Mackie A.M., Hassan K.A., Paulsen I.T., Tetu S.G. (2014). Biolog Phenotype Microarrays for Phenotypic Characterization of Microbial Cells. Methods Mol. Biol..

[43] Shamshitov A., Decorosi F., Viti C., Fornasier F., Kadžienė G., Supronienė S. (2022). Characterisation of Cellulolytic Bacteria Isolated from Agricultural Soil in Central Lithuania. Sustainability.

[44] (1999). Soil Improvers and Growing Media Determination of Organic Matter Content and Ash.

[45] (2000). Characterization of Sludges—Determination of Kjeldahl Nitrogen.

[46] Martens D.A. (2000). Plant Residue Biochemistry Regulates Soil Carbon Cycling and Carbon Sequestration. Soil Biol. Biochem..

[47] Wilson D.B. (2009). Cellulases and Biofuels. Curr. Opin. Biotechnol..

[48] Kourieh R., Bennici S., Marzo M., Gervasini A., Auroux A. (2012). Investigation of the WO 3/ZrO 2 Surface Acidic Properties for the Aqueous Hydrolysis of Cellobiose. Catal. Commun..

[49] Kumar Gupta P., Sai Raghunath S., Venkatesh Prasanna D., Venkat P., Shree V., Chithananthan C., Choudhary S., Surender K., Geetha K. (2019). An Update on Overview of Cellulose, Its Structure and Applications. Cellulose.

[50] Anderson I., Abt B., Lykidis A., Klenk H.P., Kyrpides N., Ivanova N. (2012). Genomics of Aerobic Cellulose Utilization Systems in Actinobacteria. PLoS ONE.

[51] Yan S., Xu Y., Yu X.W. (2023). Role of Cellulose Response Transporter-like Protein CRT2 in Cellulase Induction in Trichoderma Reesei. Biotechnol. Biofuels Bioprod..

[52] Hu M.L., Zha J., He L.W., Lv Y.J., Shen M.H., Zhong C., Li B.Z., Yuan Y.J. (2016). Enhanced Bioconversion of Cellobiose by Industrial Saccharomyces Cerevisiae Used for Cellulose Utilization. Front. Microbiol..

[53] Prade R.A., Ayoubi P., Zhan D., Mort A.J. (1999). Pectins, Pectinases and Plant-Microbe Interactions. Biotechnol. Genet. Eng. Rev..

[54] Mohnen D. (2008). Pectin Structure and Biosynthesis. Curr. Opin. Plant Biol..

[55] Lygin A.V., Upton J., Dohleman F.G., Juvik J., Zabotina O.A., Widholm J.M., Lozovaya V.V. (2011). Composition of Cell Wall Phenolics and Polysaccharides of the Potential Bioenergy Crop-Miscanthus. GCB Bioenergy.

[56] Vogel J. (2008). Unique Aspects of the Grass Cell Wall. Curr. Opin. Plant Biol..

[57] de Oliveira D.M., Finger-Teixeira A., Rodrigues Mota T., Salvador V.H., Moreira-Vilar F.C., Correa Molinari H.B., Craig Mitchell R.A., Marchiosi R., Ferrarese-Filho O., Dantas dos Santos W. (2015). Ferulic Acid: A Key Component in Grass Lignocellulose Recalcitrance to Hydrolysis. Plant Biotechnol. J..

[58] Dodd D., Cann I.K.O. (2009). Enzymatic Deconstruction of Xylan for Biofuel Production. GCB Bioenergy.

[59] Wong D.W.S., Chan V.J., Liao H., Zidwick M.J. (2013). Cloning of a Novel Feruloyl Esterase Gene from Rumen Microbial Metagenome and Enzyme Characterization in Synergism with Endoxylanases. J. Ind. Microbiol. Biotechnol..

[60] Duan X., Dai Y., Zhang T. (2021). Characterization of Feruloyl Esterase from Bacillus Pumilus Sk52.001 and Its Application in Ferulic Acid Production from de-Starched Wheat Bran. Foods.

[61] Liang W., Xiong T., Wang X., Deng H., Bai Y., Fan T.P., Zheng X., Cai Y. (2020). A Novel Feruloyl Esterase with High Rosmarinic Acid Hydrolysis Activity from Bacillus Pumilus W3. Int. J. Biol. Macromol..

[62] Liu P., Guo J., Miao L., Liu H. (2022). Enhancing the Secretion of a Feruloyl Esterase in Bacillus Subtilis by Signal Peptide Screening and Rational Design. Protein Expr. Purif..

[63] Uraji M., Arima J., Inoue Y., Harazono K., Hatanaka T. (2014). Application of Two Newly Identified and Characterized Feruloyl Esterases from Streptomyces Sp. in the Enzymatic Production of Ferulic Acid from Agricultural Biomass. PLoS ONE.

[64] Uraji M., Tamura H., Mizohata E., Arima J., Wan K., Ogawa K., Inoue T., Hatanaka T. (2018). Loop of Streptomyces Feruloyl Esterase Plays an Important Role in the Enzyme’s Catalyzing the Release of Ferulic Acid from Biomass. Appl. Environ. Microbiol..

[65] Kheder F., Delaunay S., Abo-Chameh G., Paris C.D., Muniglia L., Girardin M. (2009). Production and Biochemical Characterization of a Type B Ferulic Acid Esterase from Streptomyces Ambofaciens. Can. J. Microbiol..

[66] Jeffries T.W. (1983). Utilization of Xylose by Bacteria, Yeasts, and Fungi. Pentoses and Lignin.

[67] Song S., Park C. (1997). Organization and Regulation of the D-Xylose Operons in *Escherichia coli* K-12: XylR Acts as a Transcriptional Activator. J. Bacteriol..

[68] Shin J.H., Roh D.H., Heo G.Y., Joo G.J., Rhee I.K. (2001). Purification and Characterization of a Regulatory Protein XyIR in the D-Xylose Operon from *Escherichia coli*. J. Microbiol. Biotechnol..

[69] Kang M.K., Lee J., Um Y., Lee T.S., Bott M., Park S.J., Woo H.M. (2014). Synthetic Biology Platform of CoryneBrick Vectors for Gene Expression in *Corynebacterium glutamicum* and Its Application to Xylose Utilization. Appl. Microbiol. Biotechnol..

[70] Dooley D., Ryu S., Giannone R.J., Edwards J., Dien B.S., Slininger P.J., Trinh C.T. (2024). Expanded Genome and Proteome Reallocation in a Novel, Robust *Bacillus coagulans* Strain Capable of Utilizing Pentose and Hexose Sugars. mSystems.

[71] González-Hernández A.I., Scalschi L., Vicedo B., Marcos-Barbero E.L., Morcuende R., Camañes G. (2022). Putrescine: A Key Metabolite Involved in Plant Development, Tolerance and Resistance Responses to Stress. Int. J. Mol. Sci..

[72] Hamade K., Fliniaux O., Fontaine J.X., Molinié R., Petit L., Mathiron D., Sarazin V., Mesnard F. (2024). NMR and LC–MS-Based Metabolomics to Investigate the Efficacy of a Commercial Bio Stimulant for the Treatment of Wheat (*Triticum aestivum*). Metabolomics.

[73] Gunnaiah R., Kushalappa A.C., Duggavathi R., Fox S., Somers D.J. (2012). Integrated Metabolo-Proteomic Approach to Decipher the Mechanisms by Which Wheat Qtl (Fhb1) Contributes to Resistance against *Fusarium graminearum*. PLoS ONE.

[74] Mazzoli R. (2021). Current Progress in Production of Building-Block Organic Acids by Consolidated Bioprocessing of Lignocellulose. Fermentation.

[75] Godlewska-Żyłkiewicz B., Świsłocka R., Kalinowska M., Golonko A., Świderski G., Arciszewska Ż., Nalewajko-Sieliwoniuk E., Naumowicz M., Lewandowski W. (2020). Biologically Active Compounds of Plants: Structure-Related Antioxidant, Microbiological and Cytotoxic Activity of Selected Carboxylic Acids. Materials.

[76] Choi I., Son H., Baek J.H. (2021). Tricarboxylic Acid (Tca) Cycle Intermediates: Regulators of Immune Responses. Life.

[77] MacLean A., Legendre F., Appanna V.D. (2023). The Tricarboxylic Acid (TCA) Cycle: A Malleable Metabolic Network to Counter Cellular Stress. Crit. Rev. Biochem. Mol. Biol..

[78] Liu H., Jin Y., Zhang R., Ning Y., Yu Y., Xu P., Deng L., Wang F. (2023). Recent Advances and Perspectives on Production of Value-Added Organic Acids through Metabolic Engineering. Biotechnol. Adv..

[79] Xu Z., Lei P., Zhai R., Wen Z., Jin M. (2019). Recent Advances in Lignin Valorization with Bacterial Cultures: Microorganisms, Metabolic Pathways, and Bio-Products. Biotechnol. Biofuels.

[80] Varman A.M., He L., Follenfant R., Wu W., Wemmer S., Wrobel S.A., Tang Y.J., Singh S. (2016). Decoding How a Soil Bacterium Extracts Building Blocks and Metabolic Energy from Ligninolysis Provides Road Map for Lignin Valorization. Proc. Natl. Acad. Sci. USA.

[81] Schmatz R., Recous S., Aita C., Tahir M.M., Schu A.L., Chaves B., Giacomini S.J. (2017). Crop Residue Quality and Soil Type Influence the Priming Effect but Not the Fate of Crop Residue C. Plant Soil.

[82] Shrestha S., Khatiwada J.R., Kognou A.L.M., Chio C., Qin W. (2023). Biomass-Degrading Enzyme(s) Production and Biomass Degradation by a Novel Streptomyces Thermocarboxydus. Curr. Microbiol..

[83] Pronyk C., Mazza G. (2012). Fractionation of Triticale, Wheat, Barley, Oats, Canola, and Mustard Straws for the Production of Carbohydrates and Lignins. Bioresour. Technol..

[84] Melrose J. (2019). The Glucosinolates: A Sulphur Glucoside Family of Mustard Anti-Tumour and Antimicrobial Phytochemicals of Potential Therapeutic Application. Biomedicines.

[85] Kim Y.-K., Lee S.-C., Cho Y.-Y., Oh H.-J., Ko Y.H. (2012). Isolation of Cellulolytic Bacillus Subtilis Strains from Agricultural Environments. ISRN Microbiol..

[86] Manzum A.A., Mamun M.A. (2019). Isolation of *Bacillus* spp. Bacteria from Soil for Production of Cellulase. Nepal J. Biotechnol..

[87] Vu V., Farkas C., Riyad O., Bujna E., Kilin A., Sipiczki G., Sharma M., Usmani Z., Gupta V.K., Nguyen Q.D. (2022). Enhancement of the Enzymatic Hydrolysis Efficiency of Wheat Bran Using the Bacillus Strains and Their Consortium. Bioresour. Technol..

[88] Huang Z., Ni G., Zhao X., Wang F., Qu M. (2021). Characterization of a GH8 β-1,4-Glucanase from Bacillus Subtilis B111 and Its Saccharification Potential for Agricultural Straws. J. Microbiol. Biotechnol..

[89] Tohamy E.Y., El-Gamal A.D., Abouelwafa A.M. (2019). Bioconversion of Rice Straw into Bioethanol by Enzymatic Hydrolysis of Bacillus Subtilis. IOSR J. Pharm. Biol. Sci..

[90] Danso B., Ali S.S., Xie R., Sun J. (2022). Valorisation of Wheat Straw and Bioethanol Production by a Novel Xylanase- and Cellulase-Producing Streptomyces Strain Isolated from the Wood-Feeding Termite, Microcerotermes Species. Fuel.

[91] Sidar A., Voshol G.P., El-Masoudi A., Vijgenboom E., Punt P.J. (2024). Highly Variable Domain Architecture in Carbohydrate-Active Enzymes Highlights Streptomyces as Promising Resource for Rice Straw Bioconversion. Bioresour. Technol. Rep..

[92] John J.A., Selvarajan E. (2023). Genomic Analysis of Lignocellulolytic Enzyme Producing Novel Streptomyces Sp.MS2A for the Bioethanol Applications. Int. J. Biol. Macromol..

[93] Cecchini D.A., Pepe O., Pennacchio A., Fagnano M., Faraco V. (2018). Directed Evolution of the Bacterial Endo-β-1,4-Glucanase from Streptomyces Sp. G12 towards Improved Catalysts for Lignocellulose Conversion. AMB Express.

[94] Gong X., Zou H., Qian C., Yu Y., Hao Y., Li L., Wang Q., Jiang Y., Ma J. (2020). Construction of in Situ Degradation Bacteria of Corn Straw and Analysis of Its Degradation Efficiency. Ann. Microbiol..

[95] Feng H., Zhi Y., Shi W., Mao L., Zhou P. (2013). Isolation, Identification and Characterization of a Straw Degrading Streptomyces Griseorubens JSD-1. Afr. J. Microbiol. Res..

[96] El-Shatoury S., Abdulla H., Dewedar A. (2007). Factorial Design for Optimization of Rice Straw Incorporation into Soil Using Micromonospora Chalcea. Res. J. Microbiol..

[97] De Menezes A.B., Lockhart R.J., Cox M.J., Allison H.E., McCarthy A.J. (2008). Cellulose Degradation by Micromonosporas Recovered from Freshwater Lakes and Classification of These Actinomycetes by DNA Gyrase B Gene Sequencing. Appl. Environ. Microbiol..

[98] Gallagher J., Winters A., Barron N., McHale L., McHale A.P. (1996). Production of Cellulase and β-Glucosidase Activity during Growth of the Actinomycete Micromonospora Chalcae on Cellulose-Containing Media. Biotechnol. Lett..

[99] Chen S.J., Lam M.Q., Thevarajoo S., Abd Manan F., Yahya A., Chong C.S. (2020). Genome Analysis of Cellulose and Hemicellulose Degrading *Micromonospora* sp. CP22. 3 Biotech.

[100] Wu D., Wei Z., Gao X., Wu J., Chen X., Zhao Y., Jia L., Wen D. (2020). Reconstruction of Core Microbes Based on Producing Lignocellulolytic Enzymes Causing by Bacterial Inoculation during Rice Straw Composting. Bioresour. Technol..

[101] Zhou Z., Shi X., Bhople P., Jiang J., Chater C.C.C., Yang S., Perez-Moreno J., Yu F., Liu D. (2024). Enhancing C and N Turnover, Functional Bacteria Abundance, and the Efficiency of Biowaste Conversion Using Streptomyces-Bacillus Inoculation. J. Environ. Manag..

[102] Cabrera M.L., Kissel D.E., Vigil M.F. (2005). Nitrogen Mineralization from Organic Residues. J. Environ. Qual..

[103] Reichel R., Wei J., Islam M.S., Schmid C., Wissel H., Schröder P., Schloter M., Brüggemann N. (2018). Potential of Wheat Straw, Spruce Sawdust, and Lignin as High Organic Carbon Soil Amendments to Improve Agricultural Nitrogen Retention Capacity: An Incubation Study. Front. Plant Sci..

[104] Curtin D., Francis G.S., McCallum F.M. (2008). Decomposition Rate of Cereal Straw as Affected by Soil Placement. Soil Res..

[105] Shamshitov A., Kadžienė G., Pini F., Supronienė S. (2024). The Role of Tillage Practices in Wheat Straw Decomposition and Shaping the Associated Microbial Communities in Endocalcaric–Epigleyic Cambisol Soil. Biol. Fertil. Soils.

