# Supplementary material for: Phenotypic Profiling of Selected Cellulolytic Strains to Develop a Crop Residue-Decomposing Bacterial Consortium
Source: Microorganisms. 2025 Jan 17;13(1):193. doi: 10.3390/microorganisms13010193 (PMC11767528; doi:10.3390/microorganisms13010193)
Supplement: Supplementary file 1 [file microorganisms-13-00193-s001.zip › microorganisms-3420699-supplementary.pdf]

Supporting information

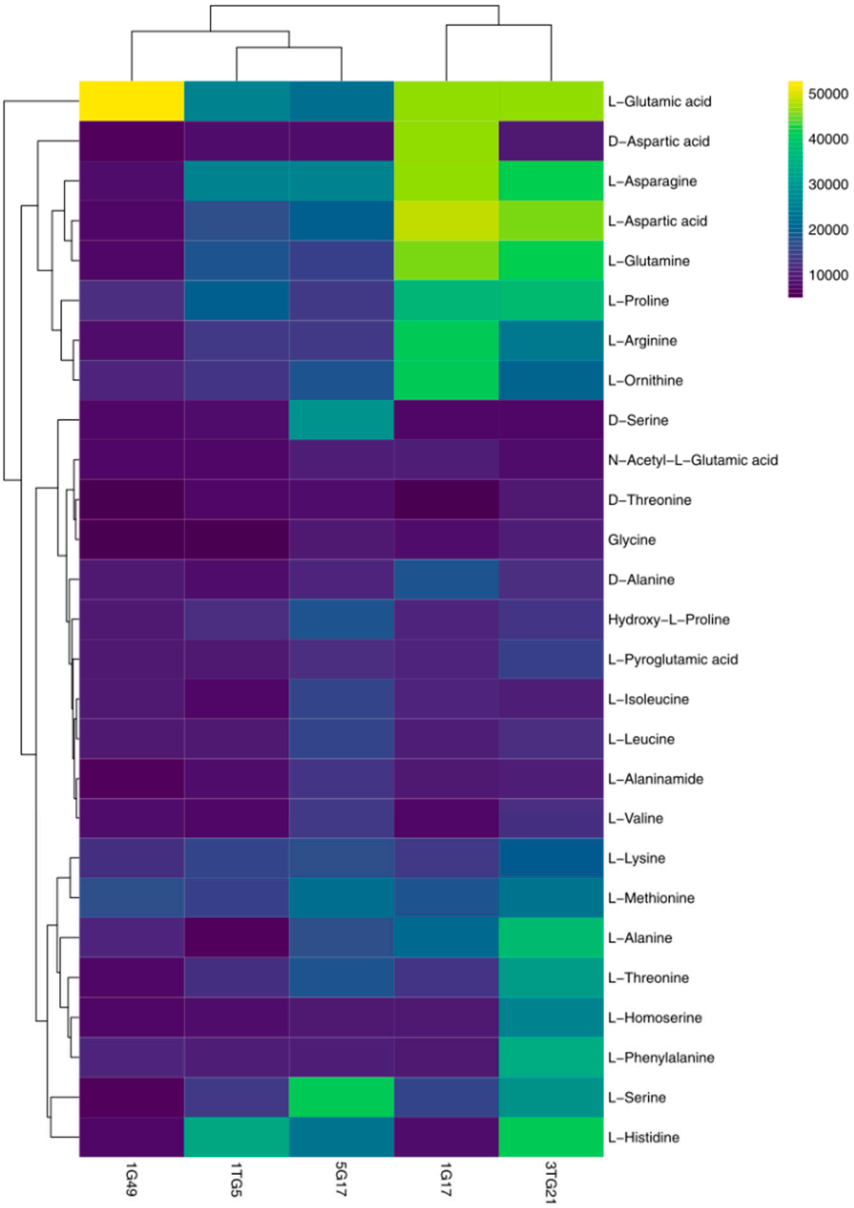

Figure S1. Heat map displaying the phenotypic utilization profiles of five bacterial strains across various amino acid sources, as assessed using Phenotype Microarray (Biolog) PM 1-2 carbon sources. Data are expressed in Arbitrary OmniLog Units (AOU) and represent the area under the kinetic curves after 60 h incubation in the OmniLog instrument at 30 °C. The color gradient ranges from purple to yellow, with purple indicating a low metabolic response or source utilization, and yellow indicating a high metabolic response or source utilization.

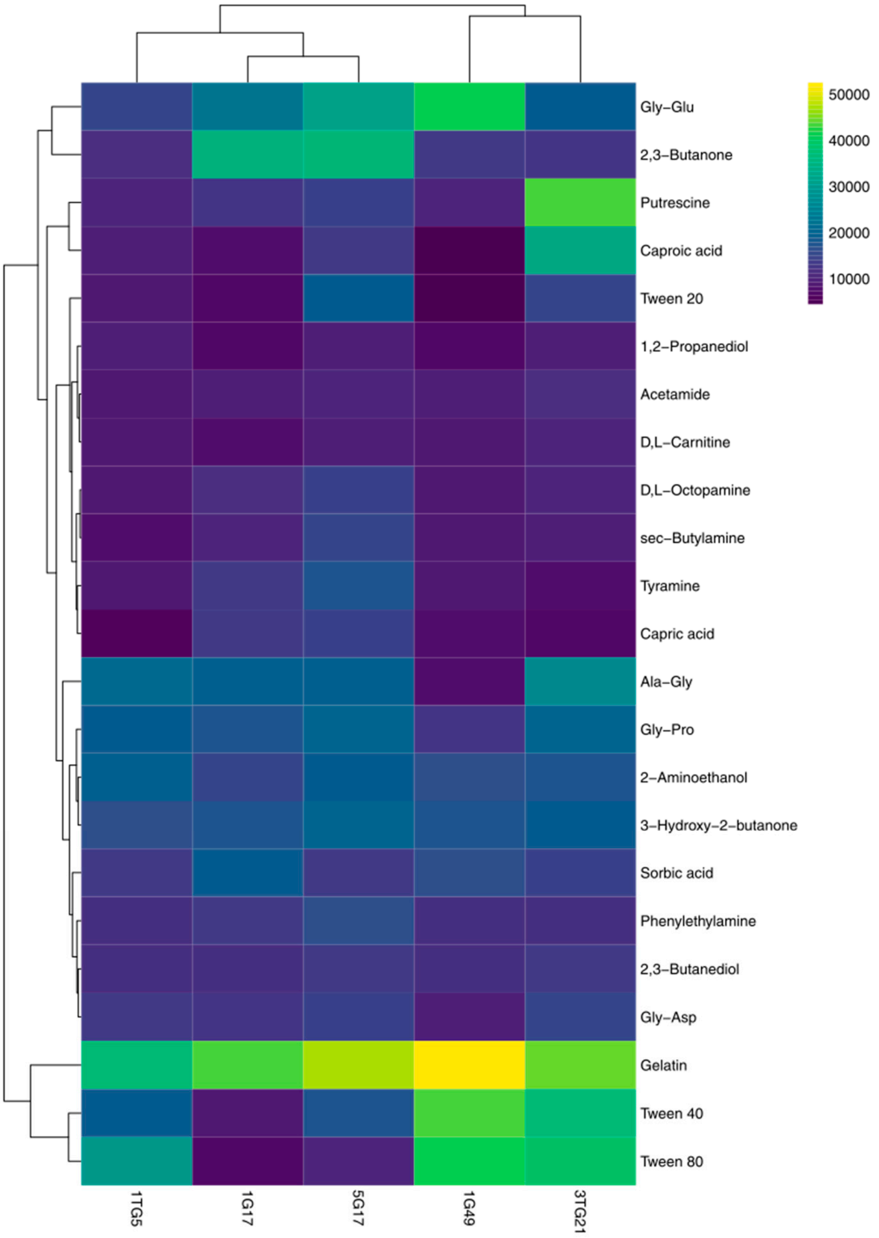

Figure S2. Heat map displaying the phenotypic utilization profiles of five bacterial strains across other sources, as assessed using Phenotype Microarray (Biolog) PM 1-2 carbon sources. Data are expressed in Arbitrary OmniLog Units (AOU) and represent the area under the kinetic curves after 60 h incubation in the OmniLog instrument at 30 °C. The color gradient ranges from purple to yellow, with purple indicating a low metabolic response or source utilization, and yellow indicating a high metabolic response or source utilization.

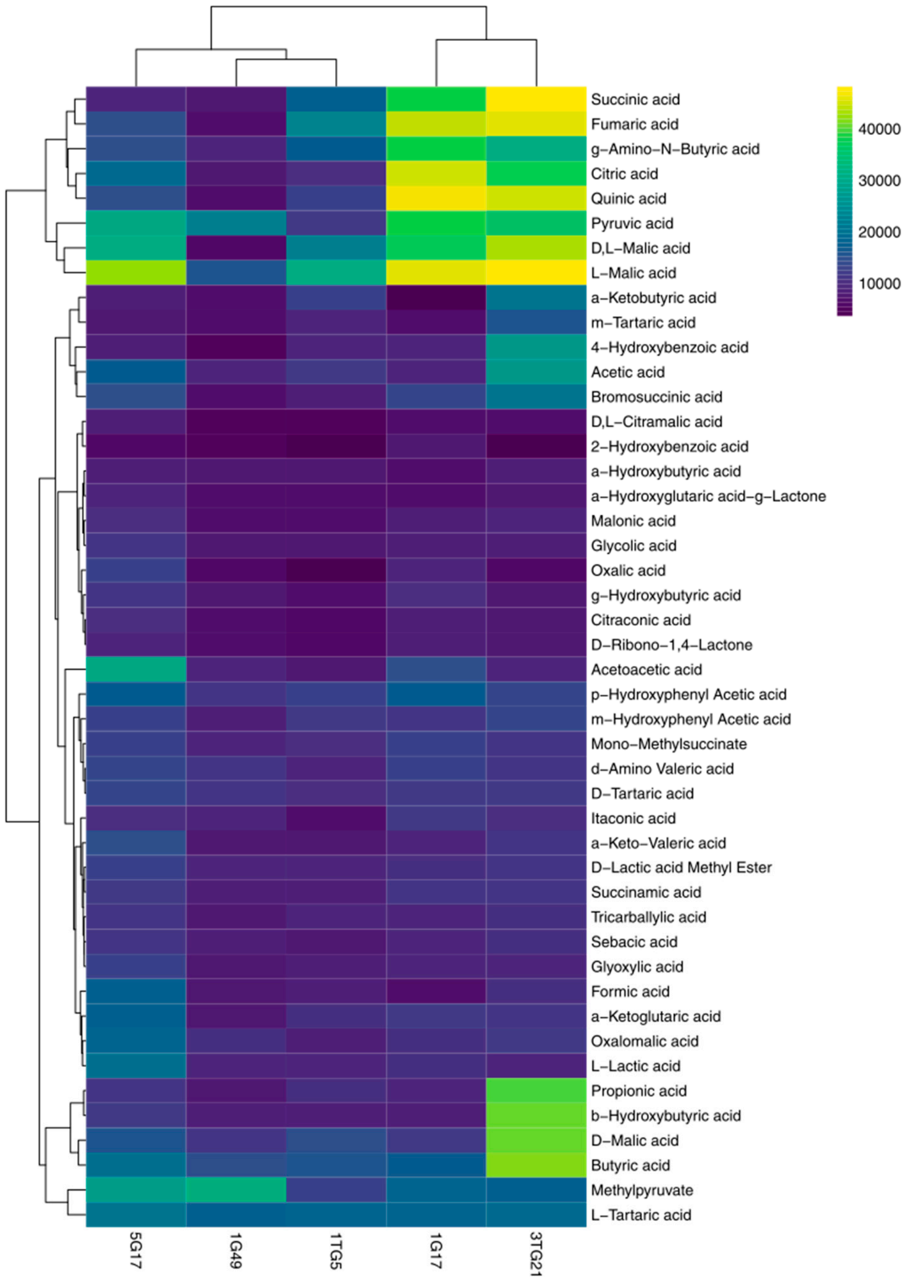

Figure S3. Heat map displaying the phenotypic utilization profiles of five bacterial strains across carboxylic acids, as assessed using Phenotype Microarray (Biolog) PM 1-2 carbon sources. Data are expressed in Arbitrary OmniLog Units (AOU) and represent the area under the kinetic curves after 60 h incubation in the OmniLog instrument at 30 °C. The color gradient ranges from purple to yellow, with purple indicating a low metabolic response or source utilization, and yellow indicating a high metabolic response or source utilization.

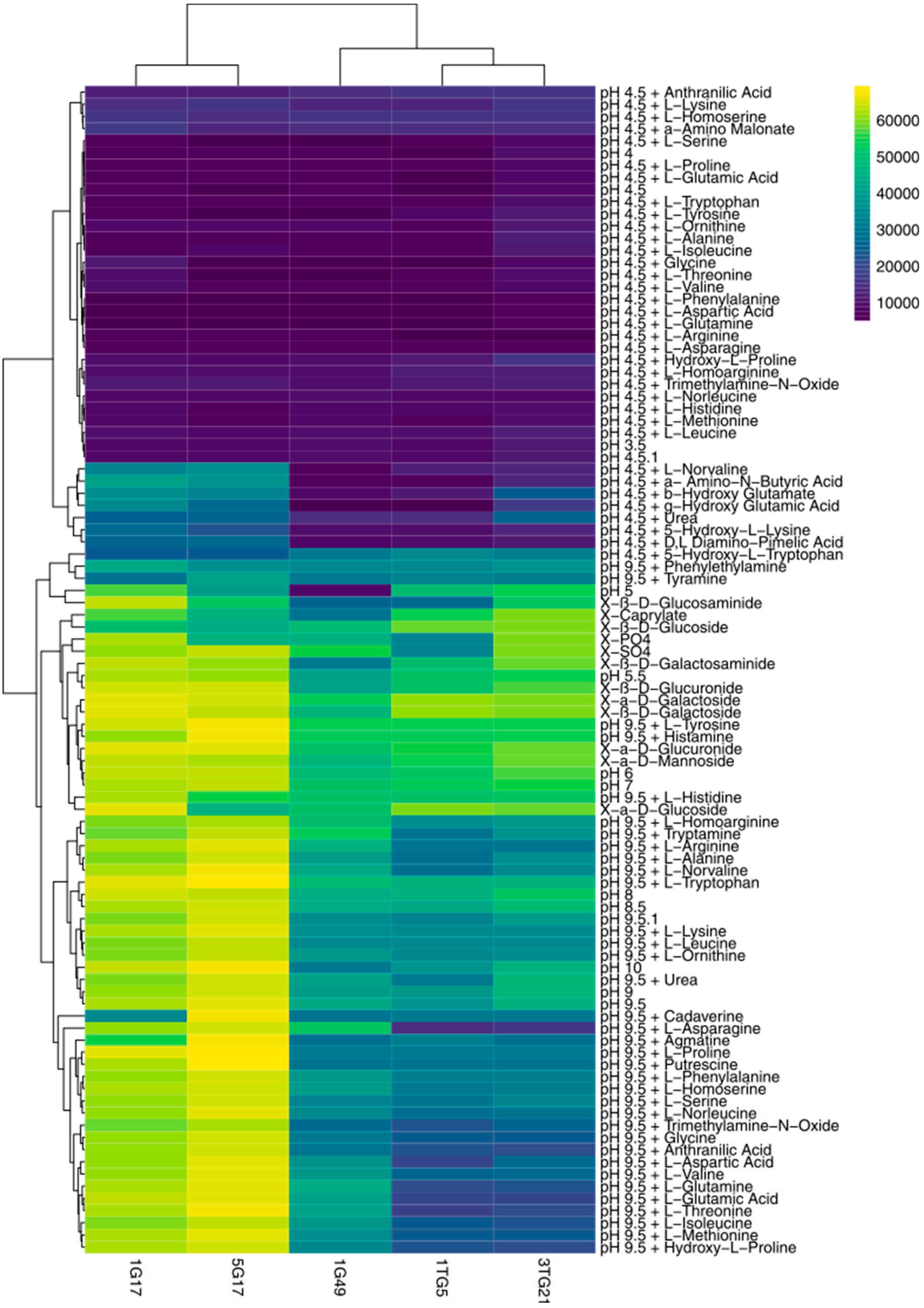

Figure S4. Heat map illustrating the pH sensitivity profiles of bacterial strains, analyzed using PM 10. Data are quantified in Arbitrary OmniLog Units (AOU), representing the area under the kinetic growth curves following 60 hours of incubation at 30°C in the OmniLog instrument. The color gradient ranges from purple to yellow, with purple indicating a low metabolic response, and yellow indicating a high metabolic response.
